# Efficacy of a novel formulation of L-Carnitine, creatine, and leucine on lean body mass and functional muscle strength in healthy older adults: a randomized, double-blind placebo-controlled study

**DOI:** 10.1186/s12986-016-0158-y

**Published:** 2017-01-18

**Authors:** Malkanthi Evans, Najla Guthrie, John Pezzullo, Toran Sanli, Roger A. Fielding, Aouatef Bellamine

**Affiliations:** 1 KGK Synergize, London, ON Canada N6A 5R8; 2 Georgetown University Medical Center, 34744, Washington, DC USA; 3 PAREXEL, 01821 Billerica, MA USA; 4 Nutrition, Exercise Physiology, and Sarcopenia Laboratory, Jean Mayer USDA Human Nutrition Research Center on Aging at Tufts University, 02111 Boston, MA USA; 5 Global Nutrition, Lonza Inc. 90 Boroline Rd, 07401 Allendale, NJ USA

**Keywords:** Sarcopenia, L-Carnitine, L-leucine, Creatine, Aging, Muscle strength, Lean body mass, Older adults, mTOR

## Abstract

**Background:**

Progressive decline in skeletal muscle mass and function are growing concerns in an aging population. Diet and physical activity are important for muscle maintenance but these requirements are not always met. This highlights the potential for nutritional supplementation. As a primary objective, we sought to assess the effect of a novel combination of L-Carnitine, creatine and leucine on muscle mass and performance in older subjects.

**Method:**

Forty-two healthy older adults aged 55–70 years were randomized to receive either a novel L-Carnitine (1500 mg), L-leucine (2000 mg), creatine (3000 mg), Vitamin D3 (10 μg) (L-Carnitine-combination) product (*n* = 14), L-Carnitine (1500 mg) (*n* = 14), or a placebo (*n* = 14) for eight weeks. We evaluated body mass by DXA, upper and lower strength by dynamometry, and walking distance by a 6-min walk test at baseline and after eight weeks of intervention. These measures, reflecting muscle mass, functional strength and mobility have been combined to generate a primary composite score. Quality of life, blood safety markers, and muscle biopsies for protein biomarker analysis were also conducted at baseline and the end of the study.

**Results:**

The primary composite outcome improved by 63.5 percentage points in the L-Carnitine-combination group vs. placebo (*P* = 0.013). However, this composite score did not change significantly in the L-Carnitine group (*P =* 0.232), and decreased slightly in the placebo group (*P =* 0.534). Participants supplemented with the L-Carnitine-combination showed a 1.0 kg increase in total lean muscle mass (*P* = 0.013), leg lean muscle mass (0.35 kg, *P =* 0.005), and a 1.0 kg increase in lower leg strength (*P* = 0.029) at week 8. In addition, these increases were significant when compared to the placebo group *(P =* 0.034, *P =* 0.026, and *P =* 0.002, respectively). Total mTOR protein expression was increased in participants in the L-Carnitine-combination group at the end of the study compared to the baseline (*P* = 0.017). This increase was also significant when compared to the placebo (*P =* 0.039), suggesting that the increase in muscle mass and strength was due to new protein synthesis and mTOR pathway activation.

**Conclusions:**

The trial did reach its primary objective. L-Carnitine combined with creatine and L-leucine significantly improved the composite score which reflects muscle mass and strength, at the end of the study compared to placebo. The combination showed an increase in mTOR protein level, a driver for increased muscle mass which translated to an improvement in muscle strength. This new combination may provide a potential nutritional intervention to promote muscle growth and improved physical functioning in older adults.

**Electronic supplementary material:**

The online version of this article (doi:10.1186/s12986-016-0158-y) contains supplementary material, which is available to authorized users.

## Background

Physical function declines with advancing age, often leading to a loss of independence and poor quality of life (QoL). Functionality is a recognized indicator of health status which is associated with declining muscle performance [1]. This age-related natural progressive decline in skeletal muscle mass and function has been termed “sarcopenia” and can eventually lead to decreased mobility and independence from pharmacological intervention [2, 3]. Strikingly, it is estimated that 45% of the older U.S. population is sarcopenic and that approximately 20% of the older U.S. population is functionally disabled, leading to upwards of $26.2 billion in healthcare expenditures annually (reviewed in [4, 5]). Several working groups established different measures to characterize sarcopenia (hand grip, gate speed, standing from sitting position, etc.). However all agreed that loss in muscle mass and functional strength leading to compromised physical activity are the common factors in defining sarcopenia [6, 7]. The mechanisms behind this condition have not been universally accepted yet [8]. However, it is well recognized that sarcopenia is accelerated by the lack of physical activity, alterations in metabolism, neuromuscular deterioration, and marginal nutrient intake and absorption [6].

Although, identifying interventions to slow down muscle wasting and loss of strength in older populations remains challenging, evidence is emerging that specific types of physical activity and nutritional intake may affect muscle loss with aging. Physical activity can improve muscle strength and function, but engaging older adults in structured resistance or endurance exercise can be arduous, especially if there are underlying health issues [9]. Growing evidence indicates that nutritional supplementation, including increased protein intake can largely reverse muscle wasting in the elderly and improve lean muscle mass and strength in older adults [10–12]. In particular, essential amino acids have been reported to increase lean body mass and basal protein synthesis [2, 13].

L-Carnitine, is a conditionally essential amino-acid-like molecule found predominantly in skeletal muscle [14], and endogenously synthesized in the liver and kidney in humans [15]. L-Carnitine is required for energy metabolism from substrates such as fat, carbohydrates and proteins [14]. Its main role is to transport long chain fatty acid to the mitochondrial matrix for β-oxidation [14]. In addition, L-Carnitine increases protein biosynthesis by sparing the use of amino acids for energy production [16]. Moreover, L-Carnitine suppressed genes responsible for protein degradation in skeletal muscle [17] and decreased muscle RING-finger protein-1 (*MuRF1*) and ubiquitin-protein conjugates, involved in protein catabolism, as well as increases the levels of IGF-1 and Akt1, two upstream elements in the mammalian target of rapamycin (mTOR), the main driver of protein anabolism [18].

L-leucine, a branched amino acid, in combination with whey protein extracts stimulated muscle protein synthesis in elderly women [19]. A similar effect was seen with the addition of L-leucine to a nutritional supplement in older and younger subjects [20]. These effects were mediated by an increase in phosphorylation of mTOR and/or its downstream substrates, p70-S6 kinase (S6K) and the Eukaryotic translation initiation factor 4E-binding protein-1 (4E-BP1) [21]. Interestingly, L-Carnitine supplementation in pigs significantly increased the bioavailability of L-leucine in a dose dependent-manner [16].

Creatine, a bioenergetic compound important in muscle metabolism, is found in meat sources and is endogenously synthesized from glycine, L-methionine and L-arginine in the liver, the kidney and the pancreas. The creatine/phosphocreatine system, responsible for maintaining intracellular ATP for immediate use during muscle contraction, is deficient in aging populations [22]. As a dietary supplement, creatine promotes muscle protein synthesis [23] and acutely enhances L-leucine bioavailability by decreasing its oxidation [24]. Creatine supplementation has also been suggested to increase the activity of the mTOR substrate, 4E-BP1, 24-h post-resistance exercise [25].

Since L-Carnitine, L-leucine, and creatine have been shown to promote muscle mass and strength [20, 26], we investigated the effect of the combination of these dietary supplements on muscle mass and strength in healthy older adults prone to sarcopenia. We developed and applied a composite score (Comp) for muscle mass, functional strength and activity as a primary outcome. QoL, as well as biomarkers of protein metabolism were assessed in an attempt to understand the mechanism of action. In addition, L-Carnitine by itself has been assessed as an exploratory endpoint.

## Methods

### Study design

An 8-week randomized, double-blind, placebo-controlled parallel study was conducted at a single center, KGK Synergize Inc., in London, ON, Canada, between January 12, 2015 and June 19, 2015. This study was reviewed by the Natural and Non-prescription Health Products Directorate (NNHPD), Health Canada and a research ethics board, which granted ethics approval in December, 2014. The study was conducted in accordance with the ethical principles that have their origins in the Declaration of Helsinki and its subsequent amendments (clinicaltrials.gov identifier NCT02317536). All participants signed an informed consent form prior to any experimental procedure.

### Participants and study assessments

Forty-two free living healthy individuals were recruited from the region of Southwestern Ontario, Canada.

The inclusion criteria were: healthy males and females between the ages 55–70 years; body mass index (BMI) of 21.0–33.0 kg/m^2^, sedentary lifestyle (defined by Stanford questionnaire, Additional file 1: Table S1), maintain current dietary habits and activity levels, if taking supplements agree to maintain dosing regimen for at least one month prior to baseline and during the study, agree not to start any new supplements, give voluntary, written informed consent to participate.

Exclusion criteria were: smokers or smoked within the past year; weight loss/gain >4.5 kg within three months of randomization; uncontrolled hypertension (>140 mmHg), renal or hepatic disease, gastrointestinal disease, pulmonary disease, or disease of the endocrine system, history of seizures, Type I and II diabetes, cancer, neurological or significant psychiatric illness, unstable thyroid disease, immunocompromised, has metal fixation plates or screws from surgery, use of oral anticoagulants, Dabigatran, or antiplatelet agents, NSAID, allergies to anesthetics, consuming >2 standard alcoholic drinks/day, sensitivity to study ingredients, participation in a previous clinical trial within 30 days of randomization.

At screening (up to 4 weeks prior to baseline), volunteers underwent a review of the inclusion/exclusion criteria, medical history and concomitant therapies, activity levels assessed, had anthropometric and vital sign measures taken, and provided fasting blood samples for safety analysis.

All eligible participants were required to complete a dual-energy X-ray absorptiometry (DXA) scan within seven days prior and were instructed to have a light meal approximately 1-h prior to their baseline visit. Randomized participants identified their stronger leg and a micro-needle muscle biopsy was performed on the weaker leg via the *Vastus Lateralis* by an experienced physician or trained delegate [27]. After the muscle biopsy the following baseline assessments were performed: upper and lower (stronger non-biopsied leg) extremity strength testing by dynamometry and the 6-min walk test, RAND SF-36 questionnaire, and Stanford exercise behavior scale. Subjects were advised to maintain their current level of activity reported during the screening. Participants were contacted by phone at week 4 to review product compliance, completion of the exercise behavior scale, concomitant therapies, adverse events and study requirements.

Participants returned after an 8-h fast, having completed a DXA scan within three days of their end of study visit at week 8. Concomitant therapies and adverse events were reviewed and blood samples were collected for safety analysis and an electrocardiogram (ECG) performed. A pre-strength tested muscle biopsy was performed approximately 1-h after consuming a meal. The 6-min walk test and upper/lower extremity strength testing using dynamometry were administered.

### Interventions

Participants consumed one sachet of either i) L-Carnitine-combination (1500 mg given as 2200 mg Carnipure® tartrate (Lonza, Switzerland), 2000 mg L-leucine, 3000 mg creatine monohydrate, and 0.01 mg (400 IU) of vitamin-D3), ii) L-Carnitine (1500 mg given as 2200 mg Carnipure® tartrate (Lonza, Switzerland)), or iii) appearance- and taste-matched placebo each day, in the morning with breakfast. Sachets were dissolved in a 300 ml bottle of orange juice. Products were manufactured by Marlyn Nutraceuticals Inc. (Phoenix, AZ, USA) and were packaged and labeled according to ICH (International Conference on Harmonisation) -GCP (Good Clinical Practice) guidelines. The investigational products were packaged in similar sized sachets.

### Randomization and blinding

Participants were randomized into three intervention groups in a 1:1:1 ratio (14 subjects per group) using block randomization by an unblinded person not involved in study assessments. Within each block of six consecutively-enrolled subjects, two subjects received placebo, two subjects received L-Carnitine, and two subjects received L-Carnitine-combination in a randomly-permuted order generated using www.randomization.com. Upon enrollment into the study, each eligible participant was assigned a randomization number based on the randomization schedule.

All clinic staff involved in product dispensing, visit assessments, conduct of the study, monitoring charts and analysis of outcomes remained blinded for the duration of the study. Supplement allocation was implemented using 6-digit randomization codes, with the list generated by an unblinded individual not involved in conducting the study. In case a serious adverse event would require the randomization code to be broken for a given participant, sealed opaque envelopes labelled with the randomization number and containing the associated supplements were prepared by the same unblinded individual, and kept at the clinical center. No premature unblinding occurred during the course of this study.

### Outcome measures

The primary composite outcome measure, Comp, assessed body mass, functional muscle strength and 6-min walk test in the L-Carnitine-combination group. As described in previous studies [28], this Comp score considered muscle strength and functional components, including the following four components: MM = muscle mass (kg), US = upper extremity strength by dynamometry (kg), LS = lower extremity strength by dynamometry (kg), 6W = 6-min walk test (meters). Comp *=* MM × US × LS × 6W. Lean body mass was determined using DXA scans (London X-ray clinic, London, ON) by trained technicians. Upper and lower body strength was evaluated via arm (Jamar-Patterson Medical, Mississauga, ON, Canada) and leg (J Tech Medical, Midvale, United States) dynamometry by the same clinical coordinator to ensure consistency. The secondary outcomes evaluated each component of the individual measurements at baseline and at week 8. The 400 m distanced walked was determined by conducting a validated 6-min walk test [29] and QoL using RAND SF-36 questionnaire. As an exploratory endpoint, L-Carnitine group was assessed for the Comp score and compared to the placebo group. Muscle biopsies were recovered from participants in all groups and subsequently used for protein analysis.

### Compliance

Compliance was assessed by counting the returned study product at each visit. Percent compliance was calculated by determining the number of dosage units consumed, divided by the number expected to have been taken and multiplied by 100. In the event of a discrepancy between information in the subject diary and the amount of study product returned, calculations were based on the product returned unless an explanation for lost product was provided. Subjects found to have a compliance of <80% or >120% at any visit were counseled. Compliance of <70% or >130% was considered as non-compliant and any subject demonstrating non-compliance for two consecutive visits was withdrawn from the study.

### Laboratory analysis

Hematology (CBC) and clinical chemistry, electrolytes (Na, K, Cl), glucose, creatinine, AST, ALT, GGT and total bilirubin were assessed by LifeLabs Medical Laboratory Services, London, Ontario, Canada.

### Micro-needle muscle biopsy

Muscle biopsy samples were obtained by an experienced physician or trained delegate as described previously [27]. In brief, participants’ legs were rested and the physician or trained delegate aseptically inserted a micro biopsy needle into the *Vastus Lateralis* muscle. The section of muscle withdrawn (~10 mg) was immediately snap-frozen in liquid nitrogen and stored at -80°C for protein analysis. The micro-needle muscle biopsy was performed on the opposite leg of that which was strength tested prior to strength testing.

### Protein analysis of biopsy samples

Muscle biopsy samples were allocated for protein determination by western blot analysis with antibodies (New England BioLabs, Mississauga, ON, Canada) against total and the phosphorylated protein targets: mTOR, phospho-mTOR (Ser2448), p70-S6K, phospho-p70-S6K (Thr389), 4E-BP1, and phospho-4E-BP1- (Thr37/46). Muscle samples were homogenized and protein concentration was determined using the Bradford method, as described previously [30]. Forty μg of protein were then loaded onto 8–12% Bolt® bis-tris plus gels (Life Technologies, Mississauga, ON, Canada) and separated by SDS-PAGE electrophoresis as previously described [31]. Detection of protein was by chemiluminescence using ECL substrate (Pierce, Waltham, MA, United States) on a ChemiGenius2 chemi-detection system (Syngene, Frederick, MD, United States).

### Sample size

Sample size was based on a standard deviation of 15%, an alpha level of 5%, 80% power, 15% attrition rate and a 16.5% detectable difference in the Comp endpoint between groups based on previous publications [32–35].

### Statistical analysis

The efficacy analysis was based on the per protocol population (PP) defined to include all subjects who had a product compliance greater than 80%, did not have any major protocol violations and completed all study procedures. Continuous numeric outcomes were tested for normality and log-normality. Log-normally distributed variables were analyzed in the logarithmic domain. Non-normal variables were analyzed by appropriate non-parametric tests.

As stated in the outcome measures, for each participant at each study visit, the composite score was calculated as the product of the (1) MM, (2) US, (3) LS, and (4) 6W (Comp = MM * US * LS * 6W). The use of a product-based composite was deemed appropriate as the relative standard deviations were approximately equal; this ensured that following a logarithmic transformation, no one component would dominate the composite. The change from baseline in the composite score was calculated as the difference between the composite score at the end of study (EOS) and the baseline (BL) composite score (ΔComp = CompEOS – CompBL). A logarithmic transformation was used to approximately normalize the composite score prior to the statistical analysis; however, the summary values are presented in the non-transformed domain.

Numeric efficacy endpoints were tested using separate linear models to compare (1) L-Carnitine to placebo and (2) L-Carnitine-combination to placebo. An analysis of covariance approach was used with the factor being the treatment group and the covariate being the value at baseline. Numerical endpoints that were intractably non-normal were assessed by the Mann-Whitney *U* test. Within group analyses of efficacy endpoints were assessed using the Student’s paired *t*-test or, in cases of intractable non-normality, the Wilcoxon Signed-Rank test. *P* ≤0.05 was considered statistically significant. Evaluations were carried out using the software package R 3.2.2 (R Core Team, 2015).

## Results

**Fig. 1:**
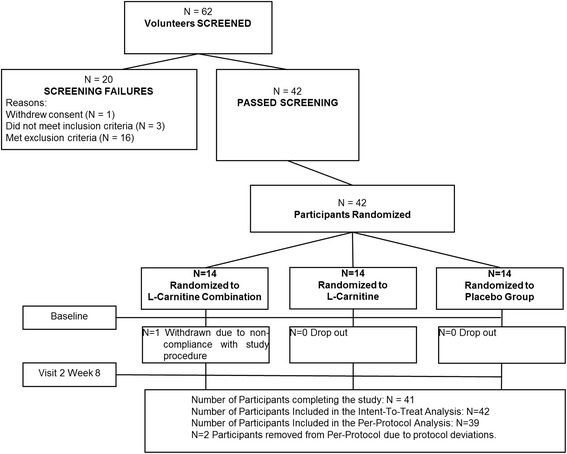
Disposition of study participants. A total of 62 participants were screened and 42 passed screening. 42 participants were enrolled in the study (14 in each group) and all but 3 (L-Carnitine group) completed the study while adhering to study protocols

The treatment groups were well matched for gender, race, activity level, use of alcohol/smoking, BMI, and weight. The placebo group had a lower average age (57.2 years) compared to the L-Carnitine group (61.4 years) and the L-Carnitine-combination group (61.1 years) (*P =* 0.006) at baseline randomization (Fig. 1 & Table 1). Although baseline levels of L-Carnitine or other active ingredients found in the L-Carnitine-combination product (L-leucine, creatine, or vitamin D) were not directly measured, for this age demographic, it has been reported in the literature that older adults may be deficient in these nutrients [36, 37]. The baseline characteristics of all participants are displayed in Table 1.

**Table 1:** Demographics and characteristics of all randomized participants

	L-Carnitine-combination *N* = 14	L-Carnitine *N* = 14	Placebo *N* = 14	*P* Value^σ^
Age (Years)				
Mean ± SD	61.1 ± 4.0 (14)	61.1 ± 4.0 (14)	57.2 ± 2.7 (14)	0.006^§^
Gender [n (%)]				
Female	9 (64%)	8 (57%)	10 (71%)	0.919
Male	5 (36%)	6 (43%)	4 (29%)	
Alcohol Use [n (%)]				
Daily	2 (14%)	0 (0%)	0 (0%)	0.787
None	4 (29%)	3 (21%)	4 (29%)	
Occasionally	4 (29%)	6 (43%)	6 (43%)	
Weekly	4 (29%)	5 (36%)	4 (29%)	
Smoking Status [n (%)]				
Ex-Smoker	0 (0%)	4 (29%)	2 (14%)	0.138
Non-Smoker	14 (100%)	10 (71%)	12 (86%)	
Race [n (%)]				
American	0 (0%)	0 (0%)	1 (7%)	0.904
Eastern European White	1 (7%)	1 (7%)	0 (0%)	
North American Indian/Aboriginal	1 (7%)	0 (0%)	0 (0%)	
South American	1 (7%)	0 (0%)	1 (7%)	
Western European White	11 (79%)	13 (93%)	12 (86%)	
Ethnicity [n (%)]				
Hispanic or Latino	1 (7%)	0 (0%)	1 (7%)	1.000
Not Hispanic or Latino	13 (93%)	14 (100%)	13 (93%)	
Regularly Exercise [n (%)]				
No	9 (64%)	10 (71%)	5 (36%)	0.218
Yes	5 (36%)	4 (29%)	9 (64%)	
Weight Change in Past 3 Months [n (%)]				
Gain	2 (14%)	0 (14%)	0 (0%)	0.679
Loss	1 (7%)	1 (7%)	2 (14%)	
No Change	11 (79%)	13 (93%)	12 (86%)	
Weight (Kg)				
Mean ± SD	76.7 ± 13.3 (14)	73.0 ± 12.9 (14)	73.7 ± 9.8 (14)	0.760
BMI (Kg/m2)				
Mean ± SD	27.71 ± 2.75 (14)	25.92 ± 3.06 (14)	26.57 ± 2.56 (14)	0.241

*N*, number, *SD* standard deviation, *Min* minimum, *Max* maximum, *n*, number, *%* percentage

^§^Between-group comparison was made using ANOVA

^σ^Between-group comparisons were made using Fisher’s Exact Test

Supplement groups with differing letter superscripts are significantly different

Probability values *P* ≤ 0.05 are statistically significant

### Compliance

Study compliance was high at >97% for all supplement arms. Overall mean compliance in the L-Carnitine-combination group was 97.4%, the L-Carnitine group was 99.2%, and the placebo group was 97.2%. Participants enrolled in the study were eligible for analysis in both the intent-to-treat (ITT) and PP analysis, with the exception of three participants in the L-Carnitine group that had completed the study out of window (*n* = 1) or had protocol deviations (*n* = 2) which excluded them from the PP analysis.

### Composite (Comp) score, primary objective

**Fig. 2:**
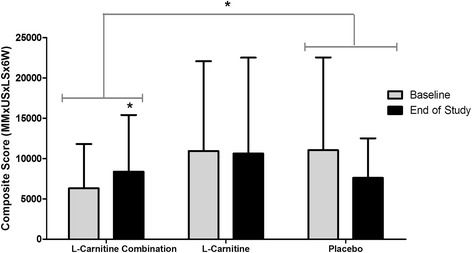
Change in the composite endpoint at baseline and end of study for participants: The Comp score was generated by multiplying the efficacy endpoints: muscle mass (MM) x upper strength (US), lower strength (LS), and 6-min walk test (6W) (x 10–3). * *P* = 0.008 with the L-Carnitine-combination group. # *P* = 0.013 between L-Carnitine-combination and placebo groups

The Comp endpoint is comprised of three critical factors that characterize sarcopenia, i) muscle mass loss, ii) muscle strength loss, and iii) physical activity. At baseline, there was no significant difference in the Comp scores between groups (*P* = 0.260). However, there was a significant absolute change (*P =* 0.008) in the Comp score for participants supplemented with L-Carnitine-combination at the end of the study, and this was greater compared to the placebo group (*P =* 0.013). When expressed as a percentage, this change for the L-Carnitine-combination translated into a 63.5 percentage point increase over placebo (Fig. 2). The effect of L-Carnitine only has been assessed as an exploratory endpoint. Participants taking L-Carnitine maintained a steady-state Comp score at the end of eight weeks relative to baseline, while the placebo group showed a non-significant reduction in Comp score at the end of the study relative to their baseline value (*P =* 0.232, Fig. 2). As an exploratory measure, the L-Carnitine Comp score was compared to placebo and no significant difference was observed between these groups (*P =* 0.576, Fig. 2).

### Muscle mass and functional strength

**Fig. 3:**
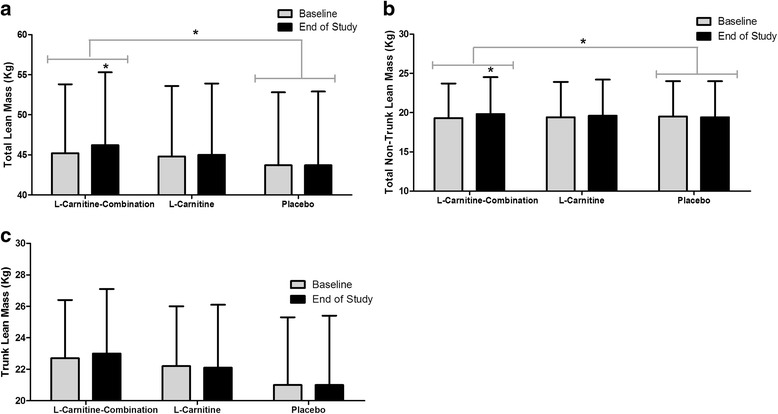
DXA body masses at baseline and end of study for participants. DXA scans were conducted at week 0 and week 8 for all participants and the following measures (Kg) were assessed (**a**), total lean mass, (**b**) total non-trunk lean mass, and (**c**) trunk lean mass. The results are expressed as the mean and SE subjects per group. * *P* < 0.05 between groups

There was a significant increase of 1.01 kg (*P =* 0.013) in total lean mass in the L-Carnitine-combination group compared to baseline, and this gain in lean mass was significantly different from the placebo group (*P =* 0.034, Fig. 3a). Total non-trunk lean mass increased significantly by 0.48 kg in the L-Carnitine-combination group by the end of the study relative to baseline (*P* = 0.006). This change was significantly greater than the placebo group (*P* = 0.016), which tended to lose total non-trunk lean mass by 0.10 kg (*P* = 0.560, Fig. 3b). Trunk lean mass did not change significantly between study arms for the duration of the study (Fig. 3c).

**Fig. 4:**
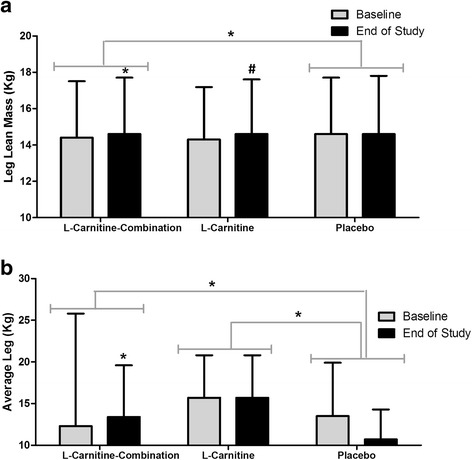
Leg mass and strength at baseline and end of study for participants. DXA scans and leg dynamometry was conducted at week 0 and week 8 for all participants and the following measures (Kg) were assessed (**a**), leg lean mass and (**b**) average leg strength. The results are expressed as the mean and SE of subjects per group. * *P* < 0.05 between groups. # *P* <0.10 trending between groups

Participants in the L-Carnitine-combination group showed a significant increase, 0.35 kg, in leg lean muscle mass (*P* = 0.005), which was significantly greater than that of the placebo group (*P* = 0.026, Fig. 4a). The L-Carnitine group showed a trend for increased leg lean muscle mass by the end of 8 weeks (*P =* 0.086, Fig. 4a). The increase in leg muscle mass translated to a significant increase in leg muscle strength by 1.0 kg for the L-Carnitine-combination group (*P =* 0.029) which was also greater than that of the placebo group (*P =* 0.002) (Fig. 4b). The leg strength in the L-Carnitine group was maintained over the course of this study and was significantly greater than the placebo group (*P =* 0.007), which exhibited a non-significant reduction in average leg strength by 2.8 kg (*P =* 0.061) after 8 weeks (Fig. 4b).

**Fig. 5:**
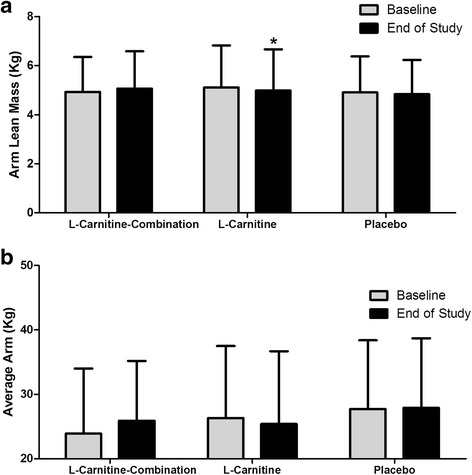
Arm mass and strength at baseline and end of study for participants. DXA scans and arm dynamometry was conducted at week 0 and week 8 for all participants and the following measures (Kg) were assessed (**a**), arm lean mass and (**b**) average arm strength. The results are expressed as the mean and SE of subjects per group. * *P* < 0.05 between groups

Participants in the L-Carnitine-combination group showed a trend towards increase in arm lean mass by 0.135 kg (*P* = 0.067) which did not translate in an improvement in functional strength as shown for the average arm grip strength (Fig. 5a). None of these changes were observed in the L-Carnitine group except for the arm lean mass which decreased by 0.123 kg (*P <* 0.001) in the L-Carnitine group at the end of 8 weeks compared to baseline (Fig. 5a). Arm strength remained similar between all supplement groups during the study (Fig. 5b).

### The 6-min walk test

No significant changes were recorded for any supplement group (Table 2).

**Table 2:** 6-min walk test at baseline and at end of the study for all participants

	L-Carnitine-combination	L-Carnitine	Placebo	*P* Value^Δ^	*P* Value^¤^
	Mean + SD (n) Within Group *P* Value	Mean + SD (n) Within Group *P* Value	Mean + SD (n) Within Group *P* Value		
Meters Walked in Six Minutes (m)					
Baseline (Week 0)	432 ± 109 (14)	458 ± 127 (11)	526 ± 80 (14)	–	–
Visit 2 (Week 8)	462 ± 113 (14)	444 ± 119 (11)	530 ± 100 (14)	–	–
Change from Baseline to Week 8	30 ± 70 (14) *P =* 0.126	−14 ± 107 (11) *P =* 0.617	3 ± 69 (14) *P =* 0.704	0. 292	0.856
Out of Breath Score Before Walking					
Baseline (Week 0)	0.21 ± 0.80 (14)	0.091 ± 0.302 (11)	0.00 ± 0.00 (14)	–	–
Visit 2 (Week 8)	0.036 ± 0.134 (14)	0.045 ± 0.151 (11)	0.000 ± 0.000 (14)	–	–
Change from Baseline to Week 8	−0.18 ± 0.67 (14) *P =* 1.000‡	−0.14 ± 0.64 (11) *P =* 0.590‡	0.00 ± 0.00 (14) *P =* 1.000‡	0. 295	0.353
Out of Breath Score After Walking					
Baseline (Week 0)	1.07 ± 2.16 (14)	0.55 ± 0.79 (11)	1.04 ± 0.91 (14)	–	–
Visit 2 (Week 8)	1.00 ± 1.79 (14)	0.41 ± 0.58 (11)	0.61 ± 0.56 (14)	–	–
Change from Baseline to Week 8	−0.07 ± 0.87 (14) *P =* 0.730‡	−0.14 ± 0.64 (11) *P* = 0.590‡	−0.43 ± 0.70 (14) ***P =*** **0.044‡**	0. 234	0.222
Change in Out of Breath Score After Walking					
Baseline (Week 0)	0.86 ± 1.60 (14)	0.45 ± 0.65 (11)	1.04 ± 0.91 (14)	–	–
Visit 2 (Week 8)	0.96 ± 1.70 (14)	0.36 ± 0.45 (11)	0.61 ± 0.56 (14)	–	–
Change from Baseline to Week 8	0.11 ± 0.68 (14) *P =* 0.792‡	−0.09 ± 0.66 (11) *P* = 0.792‡	−0.43 ± 0.70 (14) ***P =*** **0.044‡**	0. 175	0.069
Fatigue Score Before Walking					
Baseline (Week 0)	0.21 ± 0.54 (14)	0.50 ± 1.02 (11)	0.54 ± 0.93 (14)	–	–
Visit 2 (Week 8)	0.25 ± 0.80 (14)	0.18 ± 0.60 (11)	0.39 ± 0.92 (14)	–	–
Change from Baseline to Week 8	0.04 ± 0.95 (14) *P =* 0.854‡	−0.32 ± 1.27 (11) *P* = 0.461‡	−0.14 ± 0.57 (14) *P =* 0.387‡	0. 853	0.893
Fatigue Score After Walking					
Baseline (Week 0)	0.64 ± 1.36 (14)	0.82 ± 1.03 (11)	0.82 ± 0.91 (14)	–	–
Visit 2 (Week 8)	0.93 ± 1.25 (14)	0.45 ± 0.88 (11)	0.61 ± 0.86 (14)	–	–
Change from Baseline to Week 8	0.29 ± 1.17 (14) *P =* 0.394‡	−0.36 ± 1.38 (11) *P* = 0.348‡	−0.21 ± 0.64 (14) *P =* 0.266‡	0. 800	0.154
Change in Fatigue Score After Walking					
Baseline (Week 0)	0.43 ± 1.33 (14)	0.32 ± 0.64 (11)	0.29 ± 0.64 (14)	–	–
Visit 2 (Week 8)	0.68 ± 1.05 (14)	0.273 ± 0.344 (11)	0.21 ± 0.26 (14)	–	–
Change from Baseline to Week 8	0.25 ± 1.03 (14) *P =* 0.341^‡^	−0.05 ± 0.65 (11) *P* = 1.000‡	−0.07 ± 0.68 (14) *P =* 0.660‡	0. 729	0.201

*N* number, *SD* standard deviation

^Δ^Between-group comparisons for placebo and L-Carnitine were made using the Mann-Whitney *U* test

^¤^Between-group comparisons for placebo and L-Carnitine-Combination were made using the Mann-Whitney *U* test

^‡^Within-group comparisons were made using the signed-rank test

Probability values *P* ≤ 0.05 are statistically significant

The bold data represents a significant P value (P>0.05)

### Quality of life

Participants using L-Carnitine showed a significant increase in their vitality score at week 8 relative to baseline (*P =* 0.025). All other QoL measures including physical functioning, role functioning (physical or emotional), emotional well-being, social functioning, pain, and general health were not significantly altered by any supplementation (Table 3).

**Table 3:** SF-36 questionnaire results at baseline and at end of the study for all participants

	L-Carnitine-combination	L-Carnitine	Placebo	*P* Value^Δ^	*P* Value^¤^
	Mean + SD (n) Within Group *P* Value	Mean + SD (n) Within Group *P* Value	Mean + SD (n) Within Group *P* Value		
Physical Functioning					
Baseline (Week 0)	81.1 ± 19.0 (14)	86.4 ± 15.2 (11)	88.6 ± 16.2 (14)	–	–
Visit 2 (Week 8)	80.7 ± 13.4 (14)	85.9 ± 12.0 (11)	88.6 ± 14.6 (14)	–	–
Change from Baseline to Week 8	−0.4 ± 14.9 (14) *P =* 0.720	−0.5 ± 7.2 (11) *P* = 0.890	0.0 ± 8.3 (14) *P =* 1.000	0.642	0.487
Role Functioning/Physical					
Baseline (Week 0)	90.2 ± 17.1 (14)	95 ± 10 (11)	90.2 ± 19.7 (14)	–	–
Visit 2 (Week 8)	92.9 ± 16.0 (14)	90.9 ± 23.1 (11)	93.8 ± 12.7 (14)	–	–
Change from Baseline to Week 8	2.7 ± 25.6 (14) *P =* 0.833	−5 ± 15 (11) *P* = 1.000	3.6 ± 19.9 (14) *P =* 0.588	0.442	0.876
Role Functioning/Emotional					
Baseline (Week 0)	97.6 ± 6.1 (14)	100.0 ± 0.0 (11)	96.4 ± 13.4 (14)	–	–
Visit 2 (Week 8)	95.2 ± 12.1 (14)	87.9 ± 27.0 (11)	100.0 ± 0.0 (14)	–	–
Change from Baseline to Week 8	−2.4 ± 14.4 (14) *P =* 0.577	−12.1 ± 27.0 (11) *P* = 0.346	3.6 ± 13.4 (14) *P =* 1.000	0.081	0.655
Vitality					
Baseline (Week 0)	58.2 ± 20.1 (14)	68.2 ± 17.4 (11)	68.9 ± 21.0 (14)	–	–
Visit 2 (Week 8)	57.5 ± 19.3 (14)	77.3 ± 11.7 (11)	68.6 ± 16.5 (14)	–	–
Change from Baseline to Week 8	−0.7 ± 15.8 (14) *P =* 0.691	9.1 ± 10.0 (11) *P* **= 0.025**	−0.4 ± 19.6 (14) *P =* 0.780	0.081	0.833
Emotional Well-Being					
Baseline (Week 0)	76.3 ± 14.2 (14)	86.5 ± 7.4 (11)	84.9 ± 15.2 (14)	–	–
Visit 2 (Week 8)	81.1 ± 10.2 (14)	87.3 ± 12.2 (11)	84.3 ± 12.8 (14)	–	–
Change from Baseline to Week 8	4.9 ± 12.8 (14) *P =* 0.261	0.7 ± 10.1 (11) *P* = 0.509	−0.6 ± 13.8 (14) *P =* 0.670	0.260	0.305
Social Functioning					
Baseline (Week 0)	48.2 ± 8.3 (14)	50.0 ± 5.6 (11)	51.8 ± 4.5 (14)	–	–
Visit 2 (Week 8)	56.2 ± 19.5 (14)	52.3 ± 9.4 (11)	50.0 ± 0.0 (14)	–	–
Change from Baseline to Week 8	8.0 ± 20.0 (14) *P =* 0.202	2.3 ± 10.9 (11) *P* p = 0.572	−1.8 ± 4.5 (14) *P =* 0.346	0.315	0.175
Pain					
Baseline (Week 0)	77.7 ± 15.1 (14)	83.4 ± 12.3 (11)	84.1 ± 18.3 (14)	–	–
Visit 2 (Week 8)	78.0 ± 20.6 (14)	83.0 ± 15.4 (11)	84.8 ± 14.6 (14)	–	–
Change from Baseline to Week 8	0.4 ± 23.3 (14) *P =* 0.944	−0.5 ± 13.0 (11) *P* = 1.000	0.7 ± 11.0 (14) *P =* 1.000	0.673	0.744
General Health					
Baseline (Week 0)	75.7 ± 16.9 (14)	85.9 ± 14.3 (11)	85.4 ± 14.3 (14)	–	–
Visit 2 (Week 8)	77.5 ± 13.7 (14)	86.0 ± 8.2 (11)	83.2 ± 12.3 (14)	–	–
Change from Baseline toWeek 8	1.8 ± 11.9 (14) *P =* 0.662	0.1 ± 12.2 (11) *P* = 0.733	−2.1 ± 11.4 (14) *P =* 0.892	0.822	0.778

*N* number, *SD* standard deviation

^Δ^Between-group comparisons for placebo and L-Carnitine were made using the Mann-Whitney *U* test

^¤^Between-group comparisons for placebo and L-Carnitine-Combination were made using the Mann-Whitney *U* test

Within-group comparisons were made using the signed-rank test

Probability values *P* ≤ 0.05 are statistically significant

The bold data represents a significant P value (P>0.05)

### The mTOR pathway

**Fig. 6:**
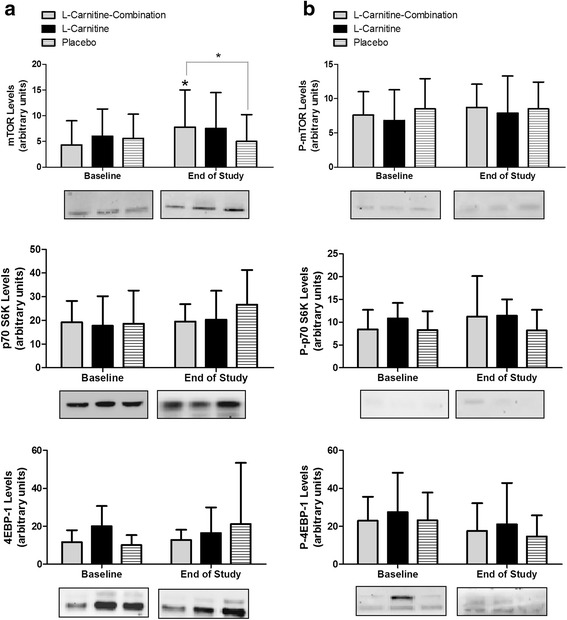
mTOR signaling proteins at baseline and end of the study for all participants. Protein samples (40 μg) obtained from participant muscle biopsies were loaded onto SDS PAGE gels and western blotting was conducted with antibodies against **a**) total; mTOR, p70 S6K, and 4E-BP1, or **b**) phosphorylated; mTOR, p70 S6K, and 4E-BP1. A representative immunoblot is shown. The graphs presented are the average densitometry values (mean and SE) of subjects. # *P* < 0.05 within group relative to baseline, * *P* < 0.05 between groups & within group relative to baseline

The mTOR pathway is the major mechanisms for protein synthesis, and because this pathway was reported to be delayed in the elderly [38], protein analysis of mTOR and its downstream effectors p70-S6K and 4EB-P1 were conducted from muscle biopsy samples (Fig. 6). L-Carnitine-combination group showed a significant increase from baseline in the total mTOR protein levels at week 8, prior to strength testing (*P =* 0.017), and this increase was 2-fold greater than that of the placebo group (*P =* 0.039) (Fig. 6a). The L-Carnitine alone showed only a marginal increase in mTOR expression without reaching significance, and the placebo group did not exhibit any change (Fig. 6a). p70-S6K and 4E-BP1 total protein was not affected by any treatment. When phosphorylation was assessed, no significant effects were observed in mTOR, nor its downstream effectors in any of the treatment groups (Fig. 6b). Other genes involved in muscle anabolism such as androgen receptor, insulin receptor, IGF-1 and its receptor were evaluated but were not affected by the supplementation (data not shown). In addition, catabolic genes *Atrogin-1* and *MuRF-1* were also not changed by the supplementation (data not shown). These results suggest that mTOR pathway is the main driver of the observed increase in muscle mass and functional strength in the L- Carnitine combination.

### Safety

Mean diastolic blood pressure was significantly increased in the L-Carnitine group compared to placebo (*P =* 0.046) at the end of study. With the exception of mean heart rate (HR) which decreased in the L-Carnitine-combination group, and mean diastolic blood pressure which increased in the L-Carnitine group, there were no significant differences in systolic blood pressure, weight, or BMI between interventions. All values for blood pressure and HR remained at a normal range for healthy adults in this age group.

The L-Carnitine-combination group showed a significant increase in creatinine concentration (*P* = 0.05), which was not unexpected given the amount of creatine present in this supplement. All other haematological parameters were within their clinical reference ranges.

### Adverse events

A total of 50 adverse events (AEs) were reported during this trial. However, of these, only three in the L-Carnitine-combination, one in the L-Carnitine group, and three in the placebo group were classified as “possibly related” to the study products.

## Discussion

Compared to individual parameters, composite biomarkers may provide a more robust method for evaluating disease progression in response to a study intervention than a single efficacy outcome [28]. In this current 8-week randomized, double-blind, placebo-controlled study, the primary composite endpoint evaluated the effect of a novel L-Carnitine-combination product on muscle mass, strength, and physical activity in older adults at risk for sarcopenia. There were significant positive changes in the composite endpoint in participants supplemented with the L-Carnitine-combination product by the end of the study, compared to the baseline. Notably, this primary Comp score was significantly greater in the L-Carnitine-combination group compared to participants in the placebo group. Interestingly, participants who supplemented with the L-Carnitine product maintained a similar Comp score from baseline to 8 weeks, while the placebo group showed a decline in Comp score. This is particularly noteworthy, as muscle strength has been reported to decline in older adults by up to 3% per year after the age of 60 [39]. This may also explain why participants in the placebo group showed a non-significant reduction in muscle strength parameters, while those in the L-Carnitine group maintained and those in the L-Carnitine-combination group improved muscle strength. In addition, clinical muscle function studies involving older men and women have observed that participants in the placebo group show declines in muscle strength over the course of the study [40, 41], relative to baseline, similar to this current report.

Our composite endpoint used multiplication of individual muscle function and strength outcomes, and fits types used in related studies. A muscular dystrophy trial by Shklyar et al., 2015 found that arithmetically derived composite scores using simple arithmetic combinations: either adding or multiplying the electrical impedance myography values and greyscale levels, were equally valid when predicting muscle function or strength parameters, such as the 6-min walk test and handheld dynamometry [28].

With respect to muscle mass and strength, this study found that the L-Carnitine-combination increased lean muscle mass by 1.0 kg. As well, lean leg mass, lower leg strength, and non-trunk lean mass improved significantly in response to an 8-week supplementation. Interestingly, physical function and QoL were not improved at the end of 8-weeks in any study arm. However, it is possible that participants receiving L-Carnitine-combination or L-Carnitine may show improvements in these measures over a longer supplementation period. To our knowledge, in addition to the whey protein effects [42], this is the only other report demonstrating that a targeted multi-nutritional supplement alone (independent of exercise) was also able to increase muscle mass among a population of older adults.

The primary Comp score for participants taking the L-Carnitine-combination product increased significantly. Though L-Carnitine alone supplementation did not improve the primary score, participants in this group showed a significant improvement in the average leg strength when compared to the placebo. One could suggest that the addition of L-leucine and creatine may have synergistic actions when incorporated with L-Carnitine. Both L-leucine and creatine were provided at sub-optimal concentrations in the L-Carnitine-combination (2000 mg and 3000 mg, respectively) than what is recommended for these supplements to optimally increase muscle mass (reviewed in [43]). Nevertheless, the combination of L-leucine, creatine, and L-Carnitine potentiated the development of muscle mass and increased strength in older adults supplemented with the L-Carnitine-combination product, likely through a common mechanism, such as promoting increase protein synthesis, increasing branched amino acid bioavailability, and decreasing protein degradation [43].

Muscle protein synthesis is stimulated by mTOR, which can be activated by amino acids and growth factors such as IGF-1 [44]. mTOR phosphorylates downstream stimulators of protein synthesis, p70 S6K and 4E-BP1, [45]. Activation of this pathway is delayed in older subjects [38], contributing to the decrease in protein synthesis with age. By the end of an 8-week supplementation with L-Carnitine-combination, total mTOR increased by 81% compared to the baseline without significantly affecting mTOR phosphorylation, as well as its downstream proteins, possibly because of the reported delay in the phosphorylation capacity of this protein kinase in the elderly [38]. An increase in total mTOR levels without changes in its phosphorylation status has been previously reported [46]. A longer supplementation period and larger sample size, may perhaps allow improved detection of mTOR phosphorylation.

In our study, the increase in total mTOR correlated with the increase in muscle mass and strength observed after supplementation with the L-Carnitine-combination. Targeting and increasing mTOR expression/activity has been proposed to attenuate age-associated sarcopenia [44]. Our data suggest a chronic effect of the L-Carnitine-combination on protein anabolism by increasing mTOR, as evidenced by increased muscle mass and functional strength. Moreover, L-Carnitine supplementation has been suggested to prevent protein catabolism [47], which is corroborated by the increase in mTOR expression observed by the end of the study.

Muscle wasting in sarcopenia populations, is associated with a shift from muscle protein synthesis to muscle protein degradation facilitated by the ubiquitin–proteasome system (UPS) [48]. *Atrogin-1* and *MuRF-1*, involved in protein degradation [49] did not change with L-Carnitine-combination supplementation, suggesting that the combination did not alter protein catabolic pathways in this study.

It is of great value that the appropriate use of dietary supplements may help reverse age-related biochemical and physiological changes leading to sarcopenia. On its own, L-Carnitine has recently been found to attenuate skeletal muscle atrophy by downregulating UPS signalling and activating mTOR [50]. While we did not observe significant changes in modulation of protein synthesis/degradation with L-Carnitine alone in this study, there is evidence that it can reverse muscle wasting under pathological conditions [51], which may have been observed with a longer study duration. Moreover, L-Carnitine supplementation has been shown to increase protein synthesis in muscle fiber and increased plasma levels of L-leucine, an essential amino acid for protein synthesis [16]. In addition, L-leucine prevented muscle degradation in clinical studies [52].

Creatine is widely used as a dietary supplement for increasing muscle mass and strength in both young and older adults [53]. A meta-analysis of creatine supplementation in the elderly concluded that creatine enhanced muscle mass, strength, and functional performance during resistance training [54]. Serum creatinine was significantly increased in the L-Carnitine-combination possibly due to the supplemental creatine. As previously stated, it is possible that L-Carnitine, creatine, and L-leucine enhanced muscle mass by synergistically increasing muscle protein synthesis.

Vitamin D3 likely had no effect based on its very low dosage in the combination (400 IU) relative to serum levels of 25(OH)D3 reported in the elderly (<40 nmol/L corresponding to 20,000 IU supplemental dose) [55]. Moreover, a recent clinical study found that vitamin D at levels ~5 times greater (48 μg) than that used in this report, had no effect on improving muscle strength in healthy older adults [56]. Despite its short duration, the current study had significant results in its composite endpoint, but did not significantly impact the physical activity (6-min walk test), QoL, and mTOR phosphorylation which may have improved with prolonged supplementation. In addition, participants in the L-Carnitine-combination group were on average older than those in the placebo group, which may account for the lower (albeit not significant) baseline values observed in the 6-min walk test. Lastly, the sample size, while adequately powered for a composite endpoint design [57], can be improved in subsequent studies based on the data from the current one.

## Conclusions

In conclusion, L-Carnitine in combination with L-leucine and creatine, significantly enhanced lean muscle mass and functional strength particularly in the lower legs, likely due to an improved protein anabolism through the mTOR pathway. The combination product was safe, well tolerated, and may provide additional performance value with prolonged use beyond the 8-week study period in healthy older adults.

## Additional file

Additional file 1: Table S1. Stanford Exercise Behavior Scale. (DOCX 13 kb)

## Abbreviations

4E-BP1: Eukaryotic translation initiation factor 4E-binding protein-1
6W: Six-minute walk test
AEs: Adverse events
BMI: Body mass index
Comp: Composite endpoint
DXA: Dual-energy X-ray absorptiometry
ITT: Intent-to-treat
LS: Lower strength
MM: Muscle mass
mTOR: Mammalian target of rapamycin
MuRF1: Muscle RING-finger protein-1
NNHPD: Natural and non-prescription health products directorate
NSAID: Non-steroidal anti-inflammatory drug
PP: Per-protocol
QoL: Quality of life
S6K: p70-S6 kinase
UPS: Ubiquitin–proteasome system
US: Upper strength
