# Supplementary material for: Efficacy of a novel formulation of L-Carnitine, creatine, and leucine on lean body mass and functional muscle strength in healthy older adults: a randomized, double-blind placebo-controlled study
Source: Nutr Metab (Lond). 2017 Jan 18;14:7. doi: 10.1186/s12986-016-0158-y (PMC5244582; doi:10.1186/s12986-016-0158-y)
Supplement: Additional file 1: Table S1. — Stanford Exercise Behavior Scale. (DOCX 13 kb) [file 12986_2016_158_MOESM1_ESM.docx]

**Supplemental Table 1: Stanford Exercise Behavior Scale**

| **How much time during the past week...** | **None** | **Less than 30 minutes/week** | **30-60 minutes/week** | **1-3 hours/week** | **More than 3 hours/week** |
| --- | --- | --- | --- | --- | --- |
| 1.   Stretching or strengthening exercises (range of motion, weights, etc.) | 0 | 1 | 2 | 3 | 4 |
| 2.   Walk for exercise | 0 | 1 | 2 | 3 | 4 |
| 3.   Swimming or aquatic exercise | 0 | 1 | 2 | 3 | 4 |
| 4.   Bicycling (including stationary exercise bikes) | 0 | 1 | 2 | 3 | 4 |
| 5. Other aerobic exercise equipment (Stairmaster, rowing, skiing machine, etc.) | 0 | 1 | 2 | 3 | 4 |
| 6. Other aerobic exercise (specify:_______) | 0 | 1 | 2 | 3 | 4 |
